# The Correlation between Metformin Use and Incident Dementia in Patients with New-Onset Diabetes Mellitus: A Population-Based Study

**DOI:** 10.3390/jpm13050738

**Published:** 2023-04-26

**Authors:** Kuang-Hua Huang, Ya-Fang Tsai, Chiachi Bonnie Lee, Shuo-Yan Gau, Tung-Han Tsai, Ning-Jen Chung, Chien-Ying Lee

**Affiliations:** 1 Department of Health Services Administration, China Medical University, Taichung 406040, Taiwan; 2 Department of Health Policy and Management, Chung Shan Medical University, Taichung 40201, Taiwan; 3 School of Medicine, Chung Shan Medical University, Taichung 40201, Taiwan; 4 Department of Pharmacology, Chung Shan Medical University, Taichung 40201, Taiwan; 5 Department of Pharmacy, Chung Shan Medical University Hospital, Taichung 40201, Taiwan

**Keywords:** dementia, metformin, diabetes mellitus, defined daily dose

## Abstract

The evidence of metformin’s effect on dementia is conflicting. This study investigates the association between metformin use and the risk of dementia among patients with diabetes mellitus (DM). This study included patients with new-onset DM between 2002 and 2013. We divided the patients into patients who used metformin and patients who did not. Two models were used to assess metformin use: the cumulative defined daily dose (cDDD) of metformin use and the intensity of metformin use. This study with 3-year and 5-year follow-ups investigated the risk of dementia among patients with DM who used metformin. At the 3-year follow-up, patients who received cDDD < 300 had an odds ratio (OR) of developing dementia of 0.92 (95% confidence interval [CI] = 0.89–0.96); patients who used metformin at intensities <10 and 10–25 DDD/month had ORs of 0.92 (95% CI: 0.87–0.97) and 0.92 (95% CI: 0.85–1.00), respectively. Metformin use at cDDD 300–500 (OR = 0.80, 95% CI = 0.56–1.15) or >500 (OR = 1.48, 95% CI = 0.48–4.60) or at an intensity >25 DDD/month (OR = 0.84, 95% CI = 0.60–1.18) were not associated with an incident of dementia. There were similar results at the 5-year follow-up. Patients with a low intensity of metformin use had a lower risk of dementia. However, higher doses of metformin with higher intensity exhibited no protective role in dementia. Prospective clinical trials are warranted to evaluate the actual underlying mechanisms between metformin dosage and the risk of dementia.

## 1. Introduction

Dementia is one of the main neurodegenerative disorders (NDs) in the elderly [1]. Alzheimer’s disease (AD) is the most common type of dementia among older adults and is characterized by progressive cognitive impairment and loss of memory, accounting for 60–80% of all cases [2]. The hippocampus is vulnerable to damage at early stages of AD.

Several studies have indicated that insulin signaling is involved in brain functions, such as cognition and memory, which are impaired in the brains of AD patients and AD experimental models. Neuronal insulin resistance (IR) is also present in the brain and can be induced by amyloid β-peptide oligomers in primary cultures of hippocampal neurons in mice and monkeys [3,4,5]. IR in diabetes mellitus (DM) and obesity has been linked with functional and structural brain change in AD brains and is associated with a risk of developing AD [6]. Evidence has indicated a clear positive association between type 2 diabetes mellitus (T2DM) and AD risk [7]; patients with T2DM are at higher risk of dementia. An epidemiological study shows that T2DM patients has been linked to a 1.5–2-fold increased risk for developing mild cognitive impairment [8]. Metformin is an orally effective and insulin-sensitizing antidiabetic medication and is the first-line treatment of T2DM for most patients [9]. Metformin has been suggested to potentially provide neuroprotective effects through the mediation of inflammatory response inhibition [10] and the improvement of cognitive function [11]. Several studies have demonstrated that T2DM patients who receive metformin exhibit a lower risk of dementia [12,13,14,15].

Several possible mechanisms have been proposed that link metformin use with the risk of dementia [16,17] [18]. However, several longitudinal studies have challenged findings on the protective role of metformin during dementia pathogenesis [19,20,21]. More studies have instead indicated that metformin use may increase the risk of dementia [16,22,23,24]. Therefore, the correlation between metformin use for T2DM and the risk of dementia warrant further research. However, few epidemiological studies have employed a nationwide database to examine the association between metformin use and the risk of dementia. We conducted a population-based study to investigate the association between metformin use and the risk of dementia in patients with T2DM.

## 2. Materials and Methods

### 2.1. Data Source

This study was based on Taiwan’s National Health Insurance Research Database (NHIRD) released by the Health and Welfare Data Science Center (HWDC) at the Ministry of Health and Welfare in Taiwan. The NHIRD contains the information of beneficiaries from the Taiwanese National Health Insurance (NHI) program and can serve as a foundation for the procurement of real-world evidence to support clinical decisions and healthcare policymaking [25,26]. The NHI is a government-run, single-payer, national social insurance program that has operated since 1995. As the NHI covers more than 99% of residents in Taiwan, we utilized the NHIRD to examine the risk of dementia among T2DM patients receiving metformin.

### 2.2. Study Subjects

**Figure 1:**
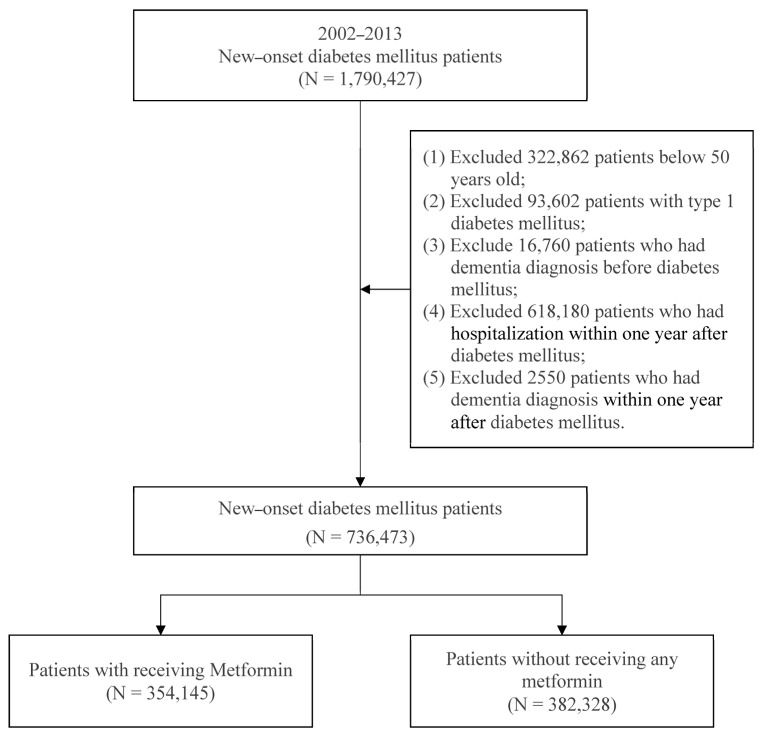
Patient selection process.

The subjects of this study were patients with new-onset DM aged ≥50 from 2002 to 2013, who were enrolled in this study. DM was defined as receiving a DM diagnosis at least three times a year, in accordance with the International Classification of Diseases, Ninth Revision, Clinical Modification [ICD-9-CM] code 250 [27], and the first DM diagnosis date was defined as the index date. Metformin was defined in accordance with the anatomical therapeutic chemical code A10BA02 and measured based on the first year after the index date. To reduce bias, we excluded the following patients: those with type 1 DM, those who had a diagnosis of dementia before DM or a diagnosis of dementia in the first year after DM, and those who were hospitalized within 1 year after DM diagnosis. A total of 736,473 new-onset patients with DM were included from 2002 to 2013. The study included 382,328 patients in the exposure group who received metformin medication within the first year after their DM diagnosis and 354,145 patients in the comparison group who did not receive any metformin medication. The selection process for study subjects is shown in Figure 1.

### 2.3. Study Design

This was a population-based study that followed patients with DM for 3 and 5 years to investigate the risk of dementia associated with metformin use. We assessed metformin intake using the defined daily dose (DDD), a standard measure of drug use and exposure. The DDD is defined by the World Health Organization as the assumed average maintenance dose per day in adults, although it may not reflect the recommended or prescribed daily dose [28]. We considered a 1-year observation period before initiating metformin treatment, following DM diagnosis, and used a baseline dose of 2 g metformin, based on the DDD [29]. The study subjects were divided into 3 groups according to the following ranges of the cumulative DDD (cDDD) of metformin: 0, <300, 300–500, and >500. In addition, the study subjects were divided into 4 groups according to the following ranges of average monthly DDD: 0, <10, 10–25, and >25. All patients were observed at 3 and 5 years after their DM diagnosis. Dementia was indicated by 3 or more outpatient visits for a dementia diagnosis within 1 year, according to the *ICD-9-CM* codes 290, 294.1, 331.0, and 331.82 and the *ICD-10-CM* codes F00-F03, F05.1, G30.0, G30.1, G30.8, and G30.9. The first dementia diagnosis date was defined as the date of incident dementia. The comorbidities consisted of hypertension (*ICD-9-CM* 401–405), hyperlipidemia (*ICD-9-CM* 272.0–272.4), hyperuricemia (*ICD-9-CM* 790.6), cerebrovascular disease (CVD; ICD-9 CM 430–438), coronary artery disease (CAD; ICD-9 CM 414.0), arrhythmia (*ICD-9-CM* 427), heart failure (*ICD-9-CM* 428.0), anxiety (*ICD-9-CM* 300.0), depression (*ICD-9-CM* 311), chronic obstructive pulmonary disease (COPD; *ICD-9-CM* 490–492, 494–496), chronic kidney disease (CKD; *ICD-9-CM* 585), obesity (*ICD-9-CM* 278.00), and alcoholism (*ICD-9-CM* 303).

### 2.4. Statistical Analysis

We conducted all analyses using SAS version 9.4, and the statistical significance level is a *p* value of <0.05. We used a chi-squared test to assess whether there were differences in baseline characteristics between patients receiving metformin and patients not receiving metformin. Multiple logistic regression with adjustments for variables was used to estimate the risk of dementia in different cDDDs of metformin use and intensities of metformin use (expressed as DDD/month). The adjusted variables in the logistic model were gender, age, income level, urbanization, DCSI, and comorbidities.

## 3. Results

### 3.1. Participant Characteristics

The average age of participants was 62.03 ± 8.76 years old (Table 1). Moreover, 51.36% of participants were women, and 48.64% were men. With regards to age groups, 64.63% were 50–64 years old, 24.66% were 65–74 years, and 10.72% were aged 75 years or older. The average age of participants who were administered metformin was 61.22 years, with a standard deviation of 8.39 years. With regard to comorbidities, 166,995 patients (43.68%) had hypertension, 68,843 patients (18.01%) had hyperlipidemia, 2821 patients (0.74%) had hyperuricemia, 17,000 patients (4.45%) had CVD, 29,886 patients (7.82%) had CAD, 14,243 patients (3.73%) had arrhythmia, 6803 patients (1.78%) had heart failure, 33,802 patients (8.84%) had anxiety, 1741 patients (0.46%) had depression, 20,361 patients (5.33%) had COPD, 1522 patients (0.40%) had CKD, 1742 patients (0.46%) had obesity, and 227 patients (0.06%) had alcoholism. Furthermore, the distribution of each comorbidity, except for alcoholism, differed significantly between patients who were and were not administered metformin (*p* < 0.001).

### 3.2. Incident Dementia among New-Onset Patients with DM Who Used Metformin

Table 2 illustrates the risk of dementia after the 3-year follow-up; a total of 7590 patients (1.03%) developed dementia in the 3 years after DM diagnosis. Among patients not treated with metformin, the incidence of dementia was 1.20%. For those treated with metformin, the incidence of dementia was 0.88% for cDDD < 300, 0.59% for cDDD between 300 and 500, and 1.28% for cDDD ≥ 500. In terms of the intensity of metformin use, the incident rate of dementia was 0.91% for DDD/month <10, 0.78% for DDD/month 10–25, and 0.62% for DDD/month >25. After adjusting relevant variables, the OR for cDDD < 300 was 0.92 (95% CI: 0.88–0.96), the OR for cDDD = 300–500 was 0.80 (95% CI: 0.56–1.15), and the OR for cDDD ≥ 500 was 1.48 (95% CI: 0.48–4.60). Patients with DM who received a DDD/month <10 and developed dementia had an OR of 0.92 (95% CI: 0.87–0.97), DDD/month 10–25 had an OR of 0.92 (95% CI: 0.85–1.00), and DDD/month >25 had an OR of 0.84 (95% CI: 0.60–1.18).

Table 3 displays the risk of incident dementia in the 5-year follow-up. After adjusting for related variables, we determined that patients with DM who received metformin at cDDD < 300, CDDD = 300–500, and cDDD ≥ 500 had ORs of developing dementia of 0.94 (95% CI: 0.91–0.97), 0.88 (95% CI: 0.70–1.12), and 1.61 (95% CI: 0.77–3.83), respectively. In terms of metformin use intensity, the ORs for dementia were 0.94 (95% CI: 0.91–0.97), 0.95 (95% CI: 0.90–1.00), and 0.92 (95% CI: 0.74–1.15) for patients who received metformin at <10, 10–25, and >25 DDD/month, respectively. In addition, the risk of dementia increased with age and the DCSI score. In terms of comorbidities, patients with CVD (OR: 1.45, 95% CI: 1.38–1.52), anxiety (OR: 1.44, 95% CI: 1.38–1.50), depression (OR: 1.58, 95% CI: 1.35–1.84), or COPD (OR: 1.08, 95% CI: 1.02–1.13) demonstrated a higher risk of dementia.

## 4. Discussion

There have been limited large-scale epidemiological studies examining the potential link between metformin intake and dementia risk among T2DM patients. However, in our study, we found that metformin use was associated with increased dementia risk among patients with T2DM. Our results indicated that patients receiving <300 cDDD of metformin and <10 and 10–25 DDD/month exhibited a reduced risk of dementia at both the 3- and 5-year follow-ups. Conversely, we observed no protective effect against dementia for cDDD > 300 and >25 DDD/month during the 3- and 5-year follow-ups among patients with T2DM. Additionally, we found that among patients with T2DM who received metformin, an increased risk of dementia was associated with older age and a higher DCSI score.

Our results indicated that patients with DM who received a metformin at cDDD < 300, <10 DDD/month, or 10–25 DDD/month exhibited a lower risk of dementia. Several mechanisms explaining the beneficial role of metformin in the prophylaxis of dementia have been proposed. Metformin may have neuroprotective effects among older patients with T2DM. Activation of adenosine monophosphate-activated protein kinase (AMPK) by metformin could partly help in realizing the minor protective effect on improving cognitive function. Metformin is an AMPK activator that suppresses hepatic glucose production and increases insulin-stimulated glucose uptake [30]. AMPK plays a major physiological role in regulating the plasticity of the hippocampal synapse and in cognitive impairment [31]. Impaired AMPK function is associated with DM and can affect neurological disorders, such as AD [32]. Metformin also plays a neuroprotective role through the AMPK/mTOR signaling pathway to control inflammatory conditions and improve the oxidative status [33]. Metformin decreased histopathological changes in AD [34], improved cognitive performance and neuronal survival in the hippocampus of animals with diabetes, and can significantly reduce neuroinflammation [35]. Metformin may also promote angiogenesis and neurogenesis in the brain [33]. Several epidemiological studies have revealed that metformin use was associated with a lower risk of dementia [13,14] and better cognitive function [15]. A meta-analysis study reported that metformin use was associated with a lower prevalence of cognitive impairment and fewer cases of incident dementia [12].

One study reported that the use of metformin for longer than 2 years was associated with a lower incidence of ND among elderly patients with T2DM; however, metformin exposure did not significantly affect the development of ND during the first 2 years [8]. Our results revealed that the protective role in dementia afforded by metformin was nonsignificant at the 3-year and 5-year follow-ups at a cDDD of 300–500 and >500 and at 10–25 and >25 DDD/month. Studies have suggested possible harmful mechanisms between metformin use and dementia risk [16,17,22]. Metformin also increased the β-amyloid accumulation [22]. However, the claim that metformin plays a protective role during dementia pathogenesis has been challenged in several longitudinal studies. One study from the United Kingdom revealed that long-term metformin use is associated with an increased risk of AD [19]. Another study indicated that metformin use can induce vitamin B12 deficiencies in a dose-dependent manner [36]. A study from Australia found that metformin-induced vitamin B12 deficiencies were associated with cognitive impairment among patients with DM [20]. A retrospective cohort study in Taiwan demonstrated that metformin use among patients with T2DM may be a risk factor for ND, including dementia and Parkinson’s disease [21]. Several studies have indicated that this effect could be due to vitamin B12 deficiencies that are potentiated by metformin usage and that contribute to cognitive impairment [18,37,38]. Several studies have expressed that metformin use was associated with lower plasma vitamin B12 levels [18,39,40]. A meta-analysis study revealed that a negative association between metformin use and vitamin B12 levels in patients with T2DM [18], and greater cumulative exposure to metformin and a longer duration of metformin treatment were associated with a higher risk of vitamin B12 deficiency [41]; a metformin dosage of >2000 mg/day increased the risk of vitamin B12 deficiency 22 times [42]. T2DM patients undergoing metformin treatment, particularly those taking metformin at a large dosage (>2000 mg/day) and for a long duration (>4 years), should be regularly screened for serum vitamin B12 levels [43]. However, the mechanism underlying vitamin B12 deficiency in patients with long-term metformin use remains unclear. Nevertheless, the proposed underlying mechanisms include an alteration in the small intestine’s motility, leading to small intestinal bacterial overgrowth and subsequent inhibition of vitamin B12 calcium-dependent intrinsic factor complex absorption [44]; malabsorption leads to a decreased serum vitamin B12 plasma level. Although metformin use may lower the risk of dementia [34,35,45], the B12 deficiencies associated with long-term metformin use and high doses of metformin exhibited no protective role in dementia. Therefore, long-term metformin use is associated with vitamin B12 deficiencies, which may counteract the potential protective benefit in dementia. Vitamin B12 deficiencies play a role in the risk of dementia in patients with T2DM who receive long-term metformin treatment and higher doses. The results of our study are consistent with those of an animal study demonstrating that a low metformin dose (100 mg/kg) may improve scopolamine-induced cognitive impairment, whereas higher doses of metformin resulted in no harmful effect [46]. A lower metformin dose may have been associated with milder DM severity, which could have affected the dementia risk. However, the actual underlying mechanisms between the metformin dosage and the risk of dementia remain unclear, and prospective clinical trials should be conducted in the future.

The DCSI is a useful tool for predicting the risk of hospitalization and mortality among patients with DM [47]. The adapted DCSI (aDCSI) has seven categories of complications and is a modified version of a risk assessment scheme that does not include medical laboratory values [47,48]. Patients with new-onset DM and with higher aDCSI scores exhibited a higher risk of developing dementia [49]. Our study indicated that patients with DM who had higher DCSI scores demonstrated a higher risk of developing dementia. The DCSI may be used as an indicator for estimating the risk of dementia.

Our findings indicated that older patients with DM exhibited a higher risk of dementia, especially among patients with DM who were above 75 years old. Aging is the largest risk factor for the development and progression of dementia. Among older patients, multiple pathologies contribute to the progression of dementia, including dementia with Lewy bodies and vascular changes [50]. Metformin-related vitamin B12 deficiencies have been documented for over 40 years [18], and both T2DM and B12 deficiencies increase with age [51].

Depression is more common among patients with cognitive impairments. Depression is a key risk factor of dementia and significant cognitive decline among patients with DM [52]. We determined that depression was associated with an increased risk of incident dementia. These findings were consistent with previous studies, in which depression was associated with accelerated cognitive decline among patients with T2DM [53]. A meta-analysis revealed that anxiety is significantly associated with an increased risk of dementia [54]. Anxiety has also been recognized as a potentially modifiable dementia risk factor. Additionally, CVD is a major cause of cognitive impairment and dementia among older patients [55]. Finally, COPD may also contribute to dementia. A systematic and meta-analysis study indicated that patients with COPD exhibit a higher risk of dementia [56]. COPD may lead to chronic hypoxemia and pulmonary encephalopathy, which may considerably affect brain dysfunction [57]. The prevalence of cognitive decline among patients with COPD was associated with the severity of the COPD case [58].

Our study has several strengths. First, our study used a population-based design. Patients were selected from the total population of Taiwan; thus, the sample was representative. We believe that the combination of the NHIRD with multiple databases could be used as a powerful research engine. The population-based design may also minimize the selection bias that is common in observational studies. Second, the characteristics of the database provided sufficient statistical power to investigate the risk of dementia among patients with T2DM and who were treated with metformin. Third, we assessed patients 3 and 5 years after their T2DM diagnosis. We categorized metformin use based on cumulative defined daily doses (cDDDs) as <300, 300–500, and >500, and based on monthly dose intensity as <10, 10–25, and >25 DDD. Additionally, we explored the influence of various comorbidities on the risk of dementia in patients with T2DM.

Our study has some limitations to note. First, we did not have access to lifestyle data, such as tobacco use, alcohol consumption, and physical activity, which may impact the development and progression of dementia. Second, the ICD-9-CM and ICD-10-CM codes used to identify T2DM severity and dementia do not provide precise information; therefore, we were unable to conduct a severity-based subgroup analysis. For instance, the NHIRD database lacks information on HbA1c. Third, using the NHIRD led to some validity concerns, such as the accuracy of diagnosis codes; the NHIRD does not contain records of medical conditions. However, the NHI implements various measures to maintain the validity and accuracy of the NHIRD. For example, the NHI randomly reviews medical charts and conducts patient interviews to verify the accuracy of diagnoses. These rigorous processes help ensure the reliability and accuracy of the NHIRD.

## 5. Conclusions

The present study did not reveal a dose–response relationship of metformin use with incident dementia in DM patients. Specifically, patients with DM who received lower doses of metformin and had a lower metformin use intensity showed a comparatively reduced risk of dementia. However, higher doses of metformin with higher intensity exhibited no protective role in dementia. The mechanisms between metformin dosage and incident dementia remain unclear. It is necessary to conduct further prospective clinical trials with adequate statistical tests to evaluate the actual underlying mechanisms between metformin dosage and the risk of dementia.

## Figures and Tables

**Table 1:** Baseline characteristics of study subjects.

Variables	Total		Metformin				
			Non-Users		Users		*p*-Value
	N	%	N	%	N	%	
Total	736,473	100.00	354,145	51.36	382,328	48.64	
Gender							
Female	378,225	51.36	187,099	52.83	191,126	49.99	<0.001
Male	358,248	48.64	167,046	47.17	191,202	50.01	
Age (year) (Mean ± SD)	62.03 ± 8.76		62.90 ± 9.06		61.22 ± 8.39		<0.001
50–64	475,964	64.63	215,283	60.79	260,681	68.18	
65–74	181,583	24.66	92,699	26.18	88,884	23.25	
75+	78,926	10.72	46,163	13.04	32,763	8.57	
Income level (NTD) ^a^							<0.001
≤21,000	381,282	51.77	187,444	52.93	193,838	50.70	
21,001–33,000	174,995	23.76	78,427	22.15	96,568	25.26	
≥33,001	180,196	24.47	88,274	24.93	91,922	24.04	
Urbanization							<0.001
Level 1	203,376	27.61	103,603	29.25	99,773	26.10	
Level 2	238,177	32.34	113,779	32.13	124,398	32.54	
Level 3	114,530	15.55	52,472	14.82	62,058	16.23	
Level 4	103,176	14.01	48,606	13.72	54,570	14.27	
Level 5	17,291	2.35	8405	2.37	8886	2.32	
Level 6	31,369	4.26	14,398	4.07	16,971	4.44	
Level 7	28,554	3.88	12,882	3.64	15,672	4.10	
DCSI score ^b^							<0.001
0	447,130	60.71	211,227	59.64	235,903	61.70	
1	157,532	21.39	75,610	21.35	81,922	21.43	
2+	131,811	17.90	67,308	19.01	64,503	16.87	
Hypertension							<0.001
No	404,777	54.96	189,444	53.49	215,333	56.32	
Yes	331,696	45.04	164,701	46.51	166,995	43.68	
Hyperlipidemia							<0.001
No	579,453	78.68	265,968	75.10	313,485	81.99	
Yes	157,020	21.32	88,177	24.90	68,843	18.01	
Hyperuricemia							<0.001
No	730,080	99.13	350,573	98.99	379,507	99.26	
Yes	6393	0.87	3572	1.01	2821	0.74	
Cerebrovascular disease							<0.001
No	699,849	95.03	334,521	94.46	365,328	95.55	
Yes	36,624	4.97	19,624	5.54	17,000	4.45	
Coronary artery disease							<0.001
No	672,668	91.34	320,226	90.42	352,442	92.18	
Yes	63,805	8.66	33,919	9.58	29,886	7.82	
Arrhythmia							<0.001
No	705,090	95.74	337,005	95.16	368,085	96.27	
Yes	31,383	4.26	17,140	4.84	14,243	3.73	
Heart failure							<0.001
No	722,542	98.11	347,017	97.99	375,525	98.22	
Yes	13,931	1.89	7128	2.01	6803	1.78	
Anxiety							<0.001
No	663,384	90.08	314,858	88.91	348,526	91.16	
Yes	73,089	9.92	39,287	11.09	33,802	8.84	
Depression							<0.001
No	732,707	99.49	352,120	99.43	380,587	99.54	
Yes	3766	0.51	2025	0.57	1741	0.46	
COPD ^b^							<0.001
No	692,329	94.01	330,362	93.28	361,967	94.67	
Yes	44,144	5.99	23,783	6.72	20,361	5.33	
Chronic kidney disease							<0.001
No	730,618	99.20	349,812	98.78	380,806	99.60	
Yes	5855	0.80	4333	1.22	1522	0.40	
Obesity							0.012
No	733,254	99.56	352,668	99.58	380,586	99.54	
Yes	3219	0.44	1477	0.42	1742	0.46	
Alcoholism							0.950
No	736,037	99.94	353,936	99.94	382,101	99.94	
Yes	436	0.06	209	0.06	227	0.06	

^a^ 1 NTD ≈ 0.03 USD. ^b^ Abbreviations: DCSI, diabetes complications severity index; COPD, chronic obstructive pulmonary disease.

**Table 2:** Risk of dementia in new-onset diabetes mellitus patients in three-year follow-up.

Variables	Three-Year Follow-Up of Incident Dementia							
	Events		Model 1			Model 2		
	N	%	OR	95% CI	*p*-Value	OR	95% CI	*p*-Value
Total	7590	1.03						
cDDD of metformin use								
Non-users	4245	1.20	1					
DDD < 300	3312	0.88	0.92	0.88–0.96	<0.001	-	-	-
DDD 300–500	30	0.59	0.80	0.56–1.15	0.235	-	-	-
DDD 500+	3	1.28	1.48	0.48–4.60	0.496	-	-	-
Intensity of metformin use								
Non-users	4245	1.20				1		
<10	2526	0.91	-	-	-	0.92	0.87–0.97	<0.001
10~25	786	0.78	-	-	-	0.92	0.85–1.00	0.037
25+	33	0.62	-	-	-	0.84	0.60–1.18	0.317
Gender								
Female	4115	1.09	1			1		
Male	3475	0.97	0.94	0.90–0.98	0.006	0.94	0.90–0.98	0.006
Age (year)								
50–64	1145	0.24	1			1		
65–74	2981	1.64	6.27	5.85–6.72	<0.001	6.27	5.85–6.72	<0.001
75+	3464	4.39	15.94	14.86–17.09	<0.001	15.94	14.86–17.09	<0.001
Income level (NTD) ^a^								
≤21,000	4884	1.28	1			1		
21,001–33,000	1402	0.80	0.90	0.85–0.96	<0.001	0.90	0.85–0.96	<0.001
≥33,001	1304	0.72	0.91	0.86–0.97	0.005	0.91	0.86–0.97	0.005
Urbanization								
Level 1	1907	0.94	1			1		
Level 2	2182	0.92	0.98	0.92–1.04	0.430	0.98	0.92–1.04	0.429
Level 3	1147	1.00	0.98	0.91–1.06	0.586	0.98	0.91–1.05	0.584
Level 4	1251	1.21	1.00	0.93–1.08	0.909	1.00	0.93–1.08	0.911
Level 5	281	1.63	1.07	0.95–1.22	0.280	1.07	0.95–1.22	0.279
Level 6	458	1.46	1.07	0.97–1.19	0.202	1.07	0.97–1.19	0.202
Level 7	364	1.27	0.99	0.89–1.11	0.883	0.99	0.89–1.11	0.884
DCSI score ^b^								
0	3382	0.76	1			1		
1	1595	1.01	1.06	1.00–1.13	0.059	1.06	1.00–1.13	0.059
2+	2613	1.98	1.44	1.35–1.53	<0.001	1.44	1.35–1.53	<0.001
Hypertension								
No	3239	0.80	1			1		
Yes	4351	1.31	0.98	0.94–1.03	0.529	0.98	0.94–1.03	0.529
Hyperlipidemia								
No	5976	1.03	1			1		
Yes	1614	1.03	0.89	0.84–0.94	<0.001	0.89	0.84–0.94	<0.001
Hyperuricemia								
No	7509	1.03	1			1		
Yes	81	1.27	1.05	0.84–1.30	0.692	1.05	0.84–1.30	0.693
Cerebrovascular disease								
No	6500	0.93	1			1		
Yes	1090	2.98	1.53	1.42–1.64	<0.001	1.53	1.42–1.64	<0.001
Coronary artery disease								
No	6535	0.97	1			1		
Yes	1055	1.65	0.91	0.84–0.97	0.007	0.91	0.84–0.97	0.007
Arrhythmia								
No	7014	0.99	1			1		
Yes	576	1.84	1.01	0.93–1.11	0.785	1.01	0.93–1.11	0.784
Heart failure								
No	7253	1.00	1			1		
Yes	337	2.42	0.95	0.85–1.07	0.379	0.95	0.85–1.07	0.379
Anxiety								
No	6331	0.95	1			1		
Yes	1259	1.72	1.50	1.41–1.59	<0.001	1.50	1.41–1.59	<0.001
Depression								
No	7496	1.02	1			1		
Yes	94	2.50	1.76	1.43–2.16	<0.001	1.76	1.43–2.16	<0.001
COPD ^b^								
No	6723	0.97	1			1		
Yes	867	1.96	1.11	1.03–1.20	0.004	1.11	1.03–1.20	0.004
Chronic kidney disease								
No	7455	1.02	1			1		
Yes	135	2.31	0.99	0.83–1.18	0.927	0.99	0.83–1.18	0.927
Obesity								
No	7573	1.03	1			1		
Yes	17	0.53	0.75	0.47–1.21	0.237	0.75	0.47–1.21	0.238
Alcoholism								
No	7583	1.03	1			1		
Yes	7	1.61	2.47	1.18–5.20	0.017	2.47	1.18–5.19	0.017

^a^ 1 NTD ≈ 0.03 USD. ^b^ Abbreviations: DCSI, diabetes complications severity index; COPD, chronic obstructive pulmonary disease.

**Table 3:** Risk of dementia in new-onset diabetes mellitus patients in five-year follow-up.

Variables	Five-Year Follow-Up of Incident Dementia							
	Events		Model 1			Model 2		
	N	%	OR	95% CI	*p*-Value	OR	95% CI	*p*-Value
Total	15,989	2.17						
cDDD of metformin use								
Non-users	8801	2.49	1					
DDD < 300	7111	1.89	0.94	0.91–0.97	<0.001	-	-	-
DDD 300–500	70	1.37	0.88	0.70–1.12	0.304	-	-	-
DDD 500+	7	2.98	1.61	0.77–3.38	0.207	-	-	-
Intensity of metformin use								
Non-users	8801	2.49				1		
<10	5409	1.96	-	-	-	0.94	0.91–0.97	<0.001
10~25	1702	1.70	-	-	-	0.95	0.90–1.00	0.035
25+	77	1.44	-	-	-	0.92	0.74–1.15	0.477
Gender								
Female	8811	2.33	1			1		
Male	7178	2.00	0.90	0.88–0.93	<0.001	0.90	0.88–0.93	<0.001
Age (year)								
50–64	2657	0.56	1			1		
65–74	6487	3.57	5.88	5.62–6.16	<0.001	5.88	5.62–6.16	<0.001
75+	6845	8.67	13.92	13.28–14.59	<0.001	13.92	13.28–14.60	<0.001
Income level (NTD) ^a^								
≤21,000	10,557	2.77	1			1		
21,001–33,000	2692	1.54	0.78	0.75–0.82	<0.001	0.78	0.75–0.82	<0.001
≥33,001	2740	1.52	0.87	0.84–0.91	<0.001	0.87	0.84–0.91	<0.001
Urbanization								
Level 1	3915	1.93	1			1		
Level 2	4633	1.95	1.01	0.97–1.05	0.731	1.01	0.97–1.05	0.733
Level 3	2397	2.09	1.00	0.95–1.05	0.997	1.00	0.95–1.05	0.994
Level 4	2720	2.64	1.07	1.02–1.13	0.006	1.07	1.02–1.13	0.007
Level 5	572	3.31	1.08	0.99–1.18	0.091	1.08	0.99–1.18	0.090
Level 6	972	3.10	1.12	1.04–1.20	0.003	1.12	1.04–1.20	0.003
Level 7	780	2.73	1.05	0.97–1.13	0.264	1.05	0.97–1.13	0.263
DCSI score ^b^								
0	7311	1.64	1			1		
1	3510	2.23	1.09	1.04–1.13	<0.001	1.09	1.04–1.13	<0.001
2+	5168	3.92	1.38	1.32–1.44	<0.001	1.38	1.32–1.44	<0.001
Hypertension								
No	6976	1.72	1			1		
Yes	9013	2.72	0.97	0.94–1.01	0.112	0.97	0.94–1.01	0.112
Hyperlipidemia								
No	12,577	2.17	1			1		
Yes	3412	2.17	0.91	0.87–0.94	<0.001	0.91	0.87–0.94	<0.001
Hyperuricemia								
No	15,821	2.17	1			1		
Yes	168	2.63	1.05	0.91–1.23	0.498	1.05	0.91–1.23	0.499
Cerebrovascular disease								
No	13,905	1.99	1			1		
Yes	2084	5.69	1.45	1.38–1.52	<0.001	1.45	1.38–1.52	<0.001
Coronary artery disease								
No	13,744	2.04	1			1		
Yes	2245	3.52	0.95	0.91–1.00	0.049	0.95	0.91–1.00	0.049
Arrhythmia								
No	14,787	2.10	1			1		
Yes	1202	3.83	1.05	0.99–1.11	0.129	1.05	0.99–1.11	0.129
Heart failure								
No	15,331	2.12	1			1		
Yes	658	4.72	0.93	0.85–1.01	0.071	0.93	0.85–1.01	0.071
Anxiety								
No	13,457	2.03	1			1		
Yes	2532	3.46	1.44	1.38–1.50	<0.001	1.44	1.38–1.50	<0.001
Depression								
No	15,820	2.16	1			1		
Yes	169	4.49	1.58	1.35–1.84	<0.001	1.58	1.35–1.84	<0.001
COPD ^b^								
No	14,266	2.06	1			1		
Yes	1723	3.90	1.08	1.02–1.13	0.004	1.08	1.02–1.13	0.004
Chronic kidney disease								
No	15,745	2.16	1			1		
Yes	244	4.17	0.92	0.81–1.05	0.194	0.92	0.81–1.05	0.195
Obesity								
No	15,943	2.17	1			1		
Yes	46	1.43	0.94	0.71–1.26	0.699	0.95	0.71–1.26	0.701
Alcoholism								
No	15,979	2.17	1			1		
Yes	10	2.29	1.69	0.91–3.14	0.098	1.69	0.91–3.14	0.098

^a^ 1 NTD ≈ 0.03 USD. ^b^ Abbreviations: DCSI, diabetes complications severity index; COPD, chronic obstructive pulmonary disease.

## Data Availability

The National Health Insurance Database used to support the findings of this study were provided by the Health and Welfare Data Science Center, Ministry of Health and Welfare (HWDC, MOHW), under license and so cannot be made freely available. Requests for access to these data should be made to HWDC (https://dep.mohw.gov.tw/dos/np-2497-113.html, accessed on 20 February 2023).
